# Autosomal recessive congenital cataracts linked to *HSF4* in a consanguineous Pakistani family

**DOI:** 10.1371/journal.pone.0225010

**Published:** 2019-12-09

**Authors:** Xiaodong Jiao, Shahid Y. Khan, Haiba Kaul, Tariq Butt, Muhammad Asif Naeem, Sheikh Riazuddin, J. Fielding Hejtmancik, S. Amer Riazuddin

**Affiliations:** 1 Ophthalmic Genetics and Visual Function Branch, National Eye Institute, National Institutes of Health, Bethesda, Maryland, United States of America; 2 The Wilmer Eye Institute, Johns Hopkins University School of Medicine, Baltimore, Maryland, United States of America; 3 National Centre of Excellence in Molecular Biology, University of the Punjab, Lahore, Pakistan; 4 Allama Iqbal Medical College, University of Health Sciences, Lahore, Pakistan; Tsinghua University School of Life Sciences, CHINA

## Abstract

**Purpose:**

To investigate the genetic basis of autosomal recessive congenital cataracts (arCC) in a large consanguineous Pakistani family.

**Methods:**

All participating members of family, PKCC074 underwent an ophthalmic examination. Slit-lamp photographs were ascertained for affected individuals that have not been operated for the removal of the cataractous lens. A small aliquot of the blood sample was collected from all participating individuals and genomic DNAs were extracted. A genome-wide scan was performed with polymorphic short tandem repeat (STR) markers and the logarithm of odds (LOD) scores were calculated. All coding exons and exon-intron boundaries of *HSF4* were sequenced and expression of *Hsf4* in mouse ocular lens was investigated. The C-terminal FLAG-tagged wild-type and mutant HSF4b constructs were prepared to examine the nuclear localization pattern of the mutant protein.

**Results:**

The ophthalmological examinations suggested that nuclear cataracts are present in affected individuals. Genome-wide linkage analyses localized the critical interval to a 10.95 cM (14.17 Mb) interval on chromosome 16q with a maximum two-point LOD score of 4.51 at θ = 0. Sanger sequencing identified a novel missense mutation: c.433G>C (p.Ala145Pro) that segregated with the disease phenotype in the family and was not present in ethnically matched controls. Real-time PCR analysis identified the expression of *HSF4* in mouse lens as early as embryonic day 15 with a steady level of expression thereafter. The immunofluorescence tracking confirmed that both wild-type and mutant HSF4 (p.Ala145Pro) proteins localized to the nucleus.

**Conclusion:**

Here, we report a novel missense mutation in *HSF4* associated with arCC in a familial case of Pakistani descent.

## Introduction

Cataract is defined as the clouding of the ocular lens and accounts for about one-third of cases of blindness in infants worldwide.[1,2] Cataracts are classified according to the morphology and/or location of opacity in the lens.[3] They compromise the nuclear, cortical, polar, or sub-capsular parts of the lens; however, in most severe cases these opacities affect the entire ocular lens. [3] Symptoms associated with cataracts include blurry vision, deteriorating color vision, and glare. Cataracts can either manifest in an isolated fashion or as one component of a syndrome affecting multiple tissues.

Approximately, one-third of cases of congenital cataract are familial that are inherited either as an autosomal dominant or an autosomal recessive trait.[4] Cataracts with diverse phenotypes, inheritance patterns and related diseases (syndromic/non-syndromic) have been associated with more than 300 genes/loci according to the Cat-Map database (http://cat-map.wustl.edu). So far, around 27 genes/loci have been associated with non-syndromic autosomal recessive cataracts including *EPHA2* (1p36.13), *GJA8* (1q21.2), *FYCO1* (3p21.31), *BFSP2* (3q22.1), 3q26.1–27.2, *GCNT2* (6p24.3–24.2), 7q21.11–31.1, AGK (7q34), 8p23.2–21.3, 9q13-22, *AKR1E2* (10p15.1), *RNLS* (10q23.31), *DNMBP* (10q24.2), *CRYAB* (11q23.1), *MIP* (12q13.3), *GJA3* (13q12.11), *HSF4* (16q22.1), *LONP1* (19p13.3), 19q13, *WDR87* (19q13.13), *SIPA1L3* (19q13.13–13.2), *LIM2* (19q13.41), *BFSP1* (20p12.1), *CRYAA* (21q22.3), *LSS* (21q22.3), *CRYBB3* (22q11.23), *CRYBB1* (22q12.1) and *CRYBA4* (22q12.1).[5–27]

HSF4 is a member of heat-shock transcription factors (HSF) DNA-binding proteins and functions to repress the expression of genes encoding heat shock proteins and molecular chaperones.[28] *HSF4* is expressed in many tissues including heart, brain, skeletal muscle, and pancreas.[28,29] The transcript consists of 13 coding exons that are alternatively spliced resulting in two different isoforms, HSF4a and HSF4b encoding for 462- and 492-amino acid polypeptides, respectively.[29] However, *Hsf4b* predominantly expressed in the murine lens essential for its development.[30]

Here, we report a consanguineous Pakistani family with four affected individuals manifesting nuclear cataracts. We localized the disease interval to chromosome 16q with the significant two-point logarithm of odds (LOD) score. Bi-directional sequencing identified a novel missense mutation in *HSF4* that segregated with the disease phenotype in the family. The immunofluorescence tracking revealed a nuclear localization pattern for the mutant HSF4 (p.Ala145Pro) and the wild-type protein.

## Materials and methods

### Clinical ascertainment

A total of >200 consanguineous Pakistani families with non-syndromic cataracts were recruited to identify new disease loci responsible for inherited visual diseases. Institutional Review Board (IRB) approval was obtained from the National Centre of Excellence in Molecular Biology, Lahore Pakistan, the National Eye Institute, and the Johns Hopkins University, Baltimore MD. The participating subjects gave informed consent consistent with the tenets of the Declaration of Helsinki. All procedures were performed in accordance with protocols approved by the IRBs of the respective institutes.

A detailed medical history was obtained by interviewing family members. Ophthalmic examinations were conducted with slit-lamp microscopy. Approximately 10 ml of blood samples were drawn from affected and unaffected members of the family and stored in 50 ml Sterilin^®^ falcon tubes containing 400 μl of 0.5 M EDTA. Blood samples were stored at -20 °C for long-term storage.

### Genomic DNA extraction

Genomic DNA was extracted from white blood cells as described previously.[14,15] Briefly, 10 ml of the blood sample was mixed with 35 ml of TE buffer (10 mM Tris-HCl, 2 mM EDTA, pH 8.0), and the TE-blood mixture was centrifuged at 2,000g for 20 minutes. The red blood cells were discarded, and the pellet was re-suspended in 35 ml of TE buffer. The TE washing was repeated two to three times and the washed pellet was re-suspended in 2 ml of TE buffer. Next, 6.25 ml of protein digestion cocktail (50 μl (10 mg ml−1) of proteinase K, 6 ml TNE buffer (10 mM Tris-HCl, 2 mM EDTA, 400 mM NaCl) and 200 μl of 10% sodium dodecyl sulfate) was added to the resuspended pellets and incubated overnight in a shaker (250 rpm) at 37 °C. The digested proteins were precipitated by adding 1 ml of 5 M NaCl, followed by vigorous shaking and chilling on ice for 15 minutes. The precipitated proteins were pelleted by centrifugation at 2,000g for 20 minutes and removed. The supernatant was mixed with equal volumes of phenol/chloroform/isoamyl alcohol (25:24:1), and the aqueous layer containing the genomic DNA was carefully collected. The DNA was precipitated with isopropanol and pelleted by centrifugation at 3,500g for 15 minutes. The DNA pellets were washed with 70% ethanol and dissolved in TE buffer. The DNA concentration was determined with a SmartSpec plus Bio-Rad Spectrophotometer (Bio-Rad, Hercules, CA).

### Genome-wide scan

A genome-wide scan was performed with 382 highly polymorphic fluorescently-labeled short tandem repeat (STR) markers from the ABI PRISM Linkage Mapping Set MD-10 (Applied Biosystems, Foster City, CA) having an average spacing of 10 cM. Multiplex polymerase chain reaction (PCR) was completed in a GeneAmp PCR System 9700 thermocycler (Applied Biosystems). Briefly, each reaction was carried out in a 5 μl mixture containing 40 ng genomic DNA, various combinations of 10 mM dye-labeled primer pairs, 0.5 ml 10× GeneAmp PCR Buffer (Applied Biosystems), 1 mM dNTP mix, 2.5 mM MgCl_2_, and 0.2 U Taq DNA polymerase (Applied Biosystems). Initial denaturation was performed for 5 minutes at 95 °C, followed by 10 cycles of 15 s at 94 °C, 15 s at 55 °C, and 30 s at 72 °C and then 20 cycles of 15 s at 89 °C, 15 s at 55 °C, and 30 s at 72 °C. The final extension was performed for 10 minutes at 72 °C. PCR products from each DNA sample were pooled and mixed with a loading cocktail containing HD-400 size standards (Applied Biosystems). The resulting PCR products were separated on an ABI 3100 DNA Analyzer (Applied Biosystems) and genotypes were assigned with GeneMapper software (Applied Biosystems).

### Linkage analysis

Two-point linkage analyses were performed using the FASTLINK version of MLINK from the LINKAGE Program Package (provided in the public domain by the Human Genome Mapping Project Resources Centre, Cambridge, UK).[31,32] The logarithm of odds (LOD) scores were calculated using ILINK. The autosomal recessive cataract was analyzed as a fully penetrant trait with an affected allele frequency of 0.001. The marker order and distances between the markers were obtained from the Marshfield database and the National Center for Biotechnology Information chromosome 16 sequence maps.

Equal allele frequencies were assumed for the initial genome-wide scan while for fine mapping allele frequencies were estimated from 96 unrelated and unaffected individuals from the Punjab province of Pakistan.

### Mutation screening

Primer pairs for individual exons of *HSF4* were designed using the primer3 software. Amplifications were performed in 25 μl reaction volume containing 50 ng of genomic DNA, 400 nM of each primer, 250 μM of dNTPs, 2.5mM MgCl_2_, and 0.2 U *Taq* DNA polymerase in the standard PCR buffer provided by the manufacturer (Applied Biosystems). PCR amplification consisted of a denaturation step at 96 °C for 5 minutes followed by 40 cycles, each consisting of 96 °C for 30 seconds followed by 57 °C for 30 seconds and 72 °C for 1 minute. PCR products were analyzed on 2% agarose gel and purified by ethanol precipitation. The PCR primers for each exon were used for bidirectional sequencing using BigDye Terminator Ready reaction mix, according to manufacturer instructions. Sequencing products were precipitated and re-suspended in 10 μl of formamide (Applied Biosystems) and denatured at 95 °C for 5 minutes. Sequencing was performed on an ABI PRISM 3100 Automated sequencer (Applied Biosystems). Sequencing results were assembled with ABI PRISM sequencing analysis software version 3.7 and analyzed with SeqScape software (Applied Biosystems).

### Real-time expression analysis

The use of mice in this study was approved by the Johns Hopkins Animal Care and Use Committee (ACUC), and all experiments were performed in accordance with a protocol approved by the Johns Hopkins ACUC. Mouse lens were obtained at different developmental time points including embryonic day 15 (E15), day 18 (E18), at birth, designated as (P0), postnatal day 3 (P3), day 6 (P6), day 9 (P9), day 12 (P12), day 14 (P14), day 21 (P21), day 28 (P28), day 42 (P42), and day 56 (P56). Mice were first anesthetized by isoflurane and subsequently euthanized through cervical dislocation. The ocular tissue was extracted, and the lenses were isolated from retina using forceps under a microscope. The lenses were divided into two pools, each representing a biological replicate for the respective developmental time point. Lenses were dissolved in Trizol reagent (Invitrogen; Carlsbad, CA) immediately after extraction and total RNA was isolated according to the manufacturer’s instructions. The quality and quantity of RNA were determined on a NanoDrop Lite spectrophotometer (Thermo Scientific, Inc.). First-strand cDNA synthesis was completed using the Superscript III kit (Invitrogen) according to the manufacturer’s instructions. Quantitative real-time PCR analyses were performed on STEP ONE ABI Real-Time PCR System using predesigned *Hsf4* TaqMan expression assays (Applied Biosystems). *Gapdh* was used as an endogenous internal control. The 2^-ΔΔCT^ method was used to determine the relative expression normalized against *Gapdh* expression at each developmental time point.

### Construction of HSF4 plasmids

The wild-type and mutant HSF4 plasmids were generated as described previously.[33] Briefly, wild-type HSF4b cDNA in pCMV6-XL4 vector (OriGene Technologies, Inc.) was digested with EcoRI and SmaI and cloned into the pFLAG-CMV-5 vector (Sigma-Aldrich, St. Louis, MO). To generate an in-frame wild-type HSF4b C-terminal FLAG tag, a 267-bp sequence containing the stop codon and the 3′ UTR was removed using a mutagenesis kit (Phusion Site-Directed Mutagenesis Kit; Thermo Fisher Scientific) following manufacturer’s protocol. The missense (p.Ala145Pro) allele of *HSF4* was introduced by site-directed mutagenesis using the QuikChange II XL mutagenesis kit (Cat. # 200521; Agilent, Inc, Santa Clara CA). The coding sequences and orientation of all constructs were confirmed by bidirectional Sanger sequencing.

### Cell culture, transfection, and immunofluorescence microscopy

HeLa (ATCC; Cat. # CCL-2) cells were grown on glass coverslips in Dulbecco’s Modified Eagle’s Medium (DMEM) with 10% fetal bovine serum (FBS) at 37 °C in a 5% CO_2_ incubator. Plasmid DNA (either the wild-type or mutant HSF4b) was diluted in 250 μl OptiMEM Reduced Serum Medium (Invitrogen) and incubated for 5 minutes at room temperature. Parallel to the above incubation, 10 μl of Lipofectamine 2000 diluted in 250 μl of OptiMEM Reduced Serum Medium was incubated for 5 minutes at room temperature. The contents of both incubations were mixed well and incubated for an additional 20 minutes at room temperature. Transfection was performed by adding a plasmid-Lipofectamine complex in a six-well plate with a glass coverslip. The cells were incubated at 37 °C in a 5% CO_2_ incubator for 24 hours.

Cells on glass coverslips were washed twice with 1 × PBS (pH 7.4) and fixed with 4% paraformaldehyde. After fixation, cells were permeabilized with PBS containing Triton X-100 (0.05%), blocked with 1% BSA (1× PBST) for 30 minutes at room temperature (RT) and incubated for 1 hour at RT with 10 μg/ml of mouse monoclonal FLAG antibody (Sigma-Aldrich) in 1% BSA (1× PBST) followed by an incubation with secondary antibody 1:100 goat anti-mouse (DyLight 488; Abcam, Cambridge, England) in the dark. The DNA was stained with DAPI (4′, 6-diamidino-2-phenylindole; Invitrogen), slides were mounted with mounting medium (VECTASHIELD; Vector Laboratories, Burlingame, CA) and were visualized with a fluoroscope (Nikon Eclipse 600; Nikon Corp., Tokyo, Japan).

## Results

**Fig 1:**
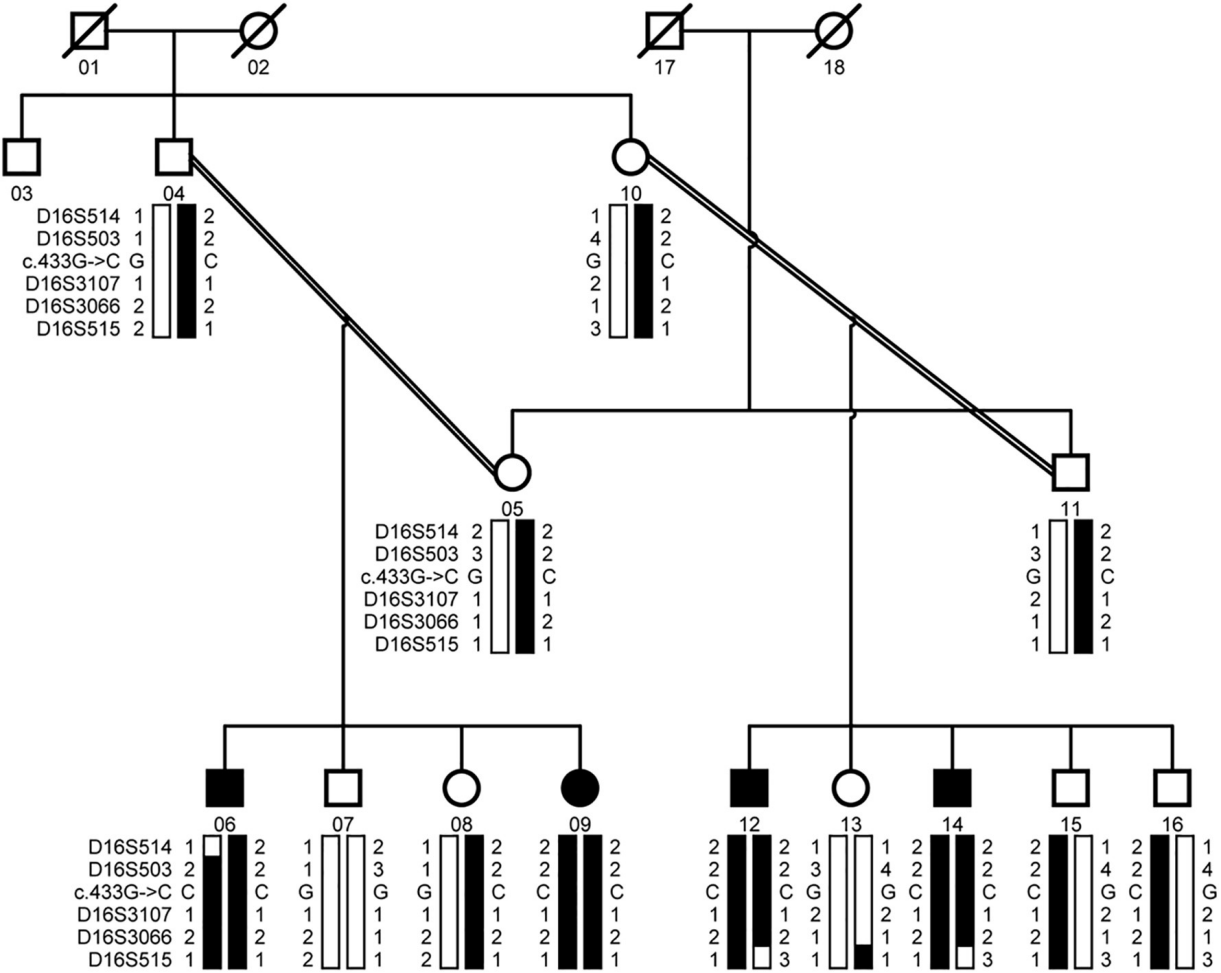
Pedigree drawing of family PKCC074. The haplotypes of five adjacent chromosome 16q markers are shown with alleles forming the risk haplotype shaded black and alleles not co-segregating with cataract are shown in white. Squares: males; circles: females; filled symbols: affected individuals; the double line between individuals: consanguineous matting; and a diagonal line through a symbol: deceased individual.

**Fig 2:**
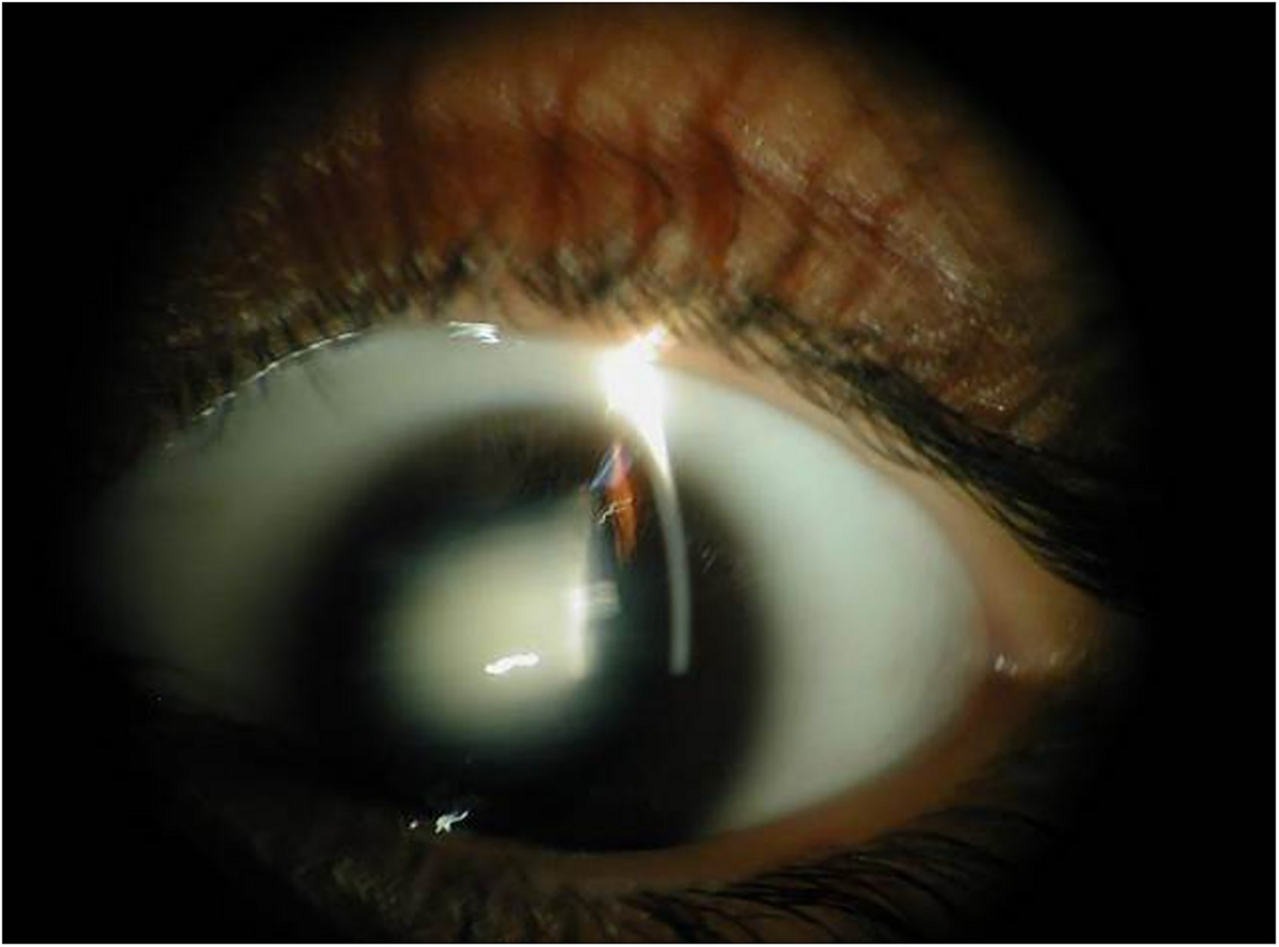
Slit-lamp photograph of individual 12 of family PKCC074. The photograph illustrates nuclear cataracts in the affected individual.

A large consanguineous family, PKCC074 with a history of congenital cataracts was recruited from the Punjab province of Pakistan (Fig 1). We enrolled a total of thirteen individuals including four individuals with cataracts from two consanguineous marriages. A detailed medical history was obtained by interviewing family members especially the parents of affected individuals, which revealed that cataracts in all four affected individuals were first observed in the first year after birth suggesting an early perhaps a congenital onset of the disease phenotype. Clinical examination conducted with slit-lamp microscopy revealed nuclear cataracts in individual 12 (Fig 2). No other ocular or systemic abnormalities were present in the family.

A genome-wide scan was completed and two-point LOD scores were calculated to localize the disease interval. Peaks of significant linkage were observed on chromosome 16q with a maximum two-point LOD score of 4.51 at θ = 0 with marker D16S503 (Table 1). Subsequently, additional STR markers in close proximity of D16S503 and D16S515 were selected from the ABI MD-5 panel. D16S3107 and D16S3066 yielded two-point LOD scores of 2.36, and 2.46 at θ = 0, respectively (Table 1). No significant two-point scores other than with chromosome 16q were observed during the genome-wide scan.

**Table 1:** Two-point LOD scores of chromosome 16q markers for family PKCC074. The asterisk indicates the marker included in the genome-wide scan.

Marker	cM	Mb	0.00	0.01	0.05	0.09	0.10	0.20	0.30	*Z*_max_	θ_max_
**D16S514**	81.15	62.30	-∞	0.81	1.83	1.95	1.94	1.55	0.95	1.95	0.09
D16S503*	83.55	63.56	4.51	4.46	4.06	3.65	3.54	2.48	1.43	4.56	0.00
**D16S3107**	85.94	67.62	2.36	2.31	2.11	1.91	1.86	1.34	0.84	2.36	0.00
**D16S3066**	89.63	73.29	2.46	2.39	2.18	1.97	1.96	1.44	0.94	2.46	0.00
D16S515*	92.10	76.48	-∞	2.31	2.04	1.79	1.72	1.11	0.58	2.37	0.00

Visual inspection of the haplotype supports the results of linkage analyses and confirms linkage to chromosome 16q (Fig 1). There is proximal recombination in individual 06 at marker D16S514. Similarly, there is distal recombination in affected individuals 12 and 14, and unaffected individual 13 at D16S515. Taken together, this places the pathogenic mutation in a 10.95 cM (14.17 Mb) interval flanked by markers D16S514, proximally and D16S515, distally (Fig 1). Alleles for markers D16S503, D16S3107, and D16S3066 are homozygous in all affected individuals (Fig 1).

**Fig 3:**
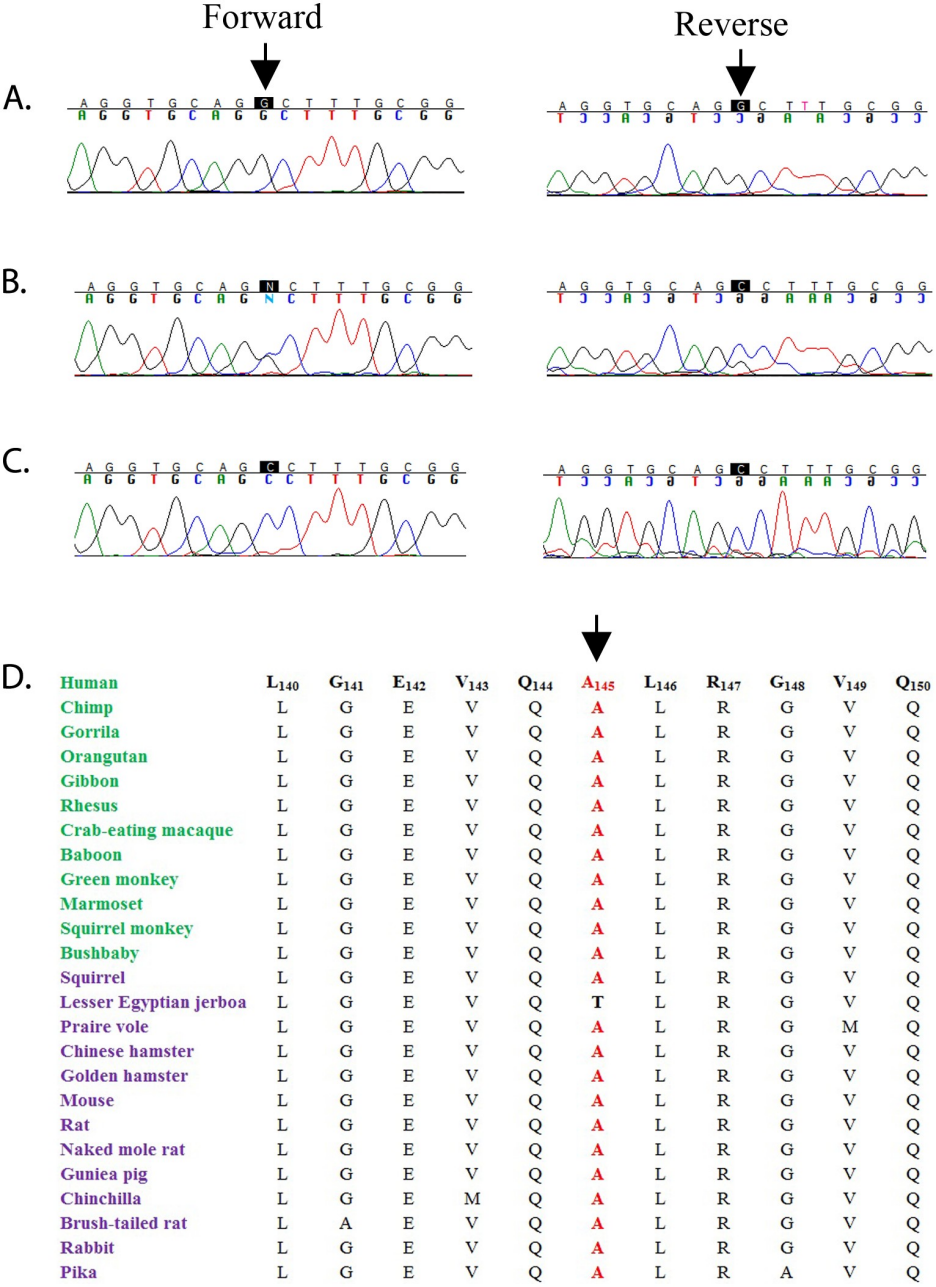
Sequence chromatograms of *HSF4* in family PKCC074. **A)** Unaffected individual 7 homozygous for wild-type allele; **B)** unaffected individual 8 heterozygous and **C)** affected individual 9, homozygous for c.433G>C variation that results in a p.Ala415Pro. **D)** Sequence alignment of amino acids illustrating the conservation of the Ala145 and its neighboring amino acids. The organisms in green and purple are primates and placental mammals respectively.

We sequenced all coding exons and exon-intron boundaries of *HSF4* and identified a missense variation: c.433G>C that leads to Proline substitution for Alanine at position 145 (p.Ala145Pro). All affected individuals in PKCC074 were homozygous for this variation whereas unaffected individuals were either heterozygous or homozygous for the wild-type allele (Fig 3A–3C). This variation was not found in 384 and 24 control chromosomes of Pakistani and Saudi descent, respectively. Additionally, this variation was not present in the 1000 genomes, the NHLB1 Exome variant server, and the dbSNP databases. We examined the evolutionary conservation of Ala145 by aligning HSF4 orthologs, which illustrated that not only Ala145 but also amino acids in the immediate neighborhood of Ala145 are well conserved among other HSF4 orthologs (Fig 3D).

**Fig 4:**
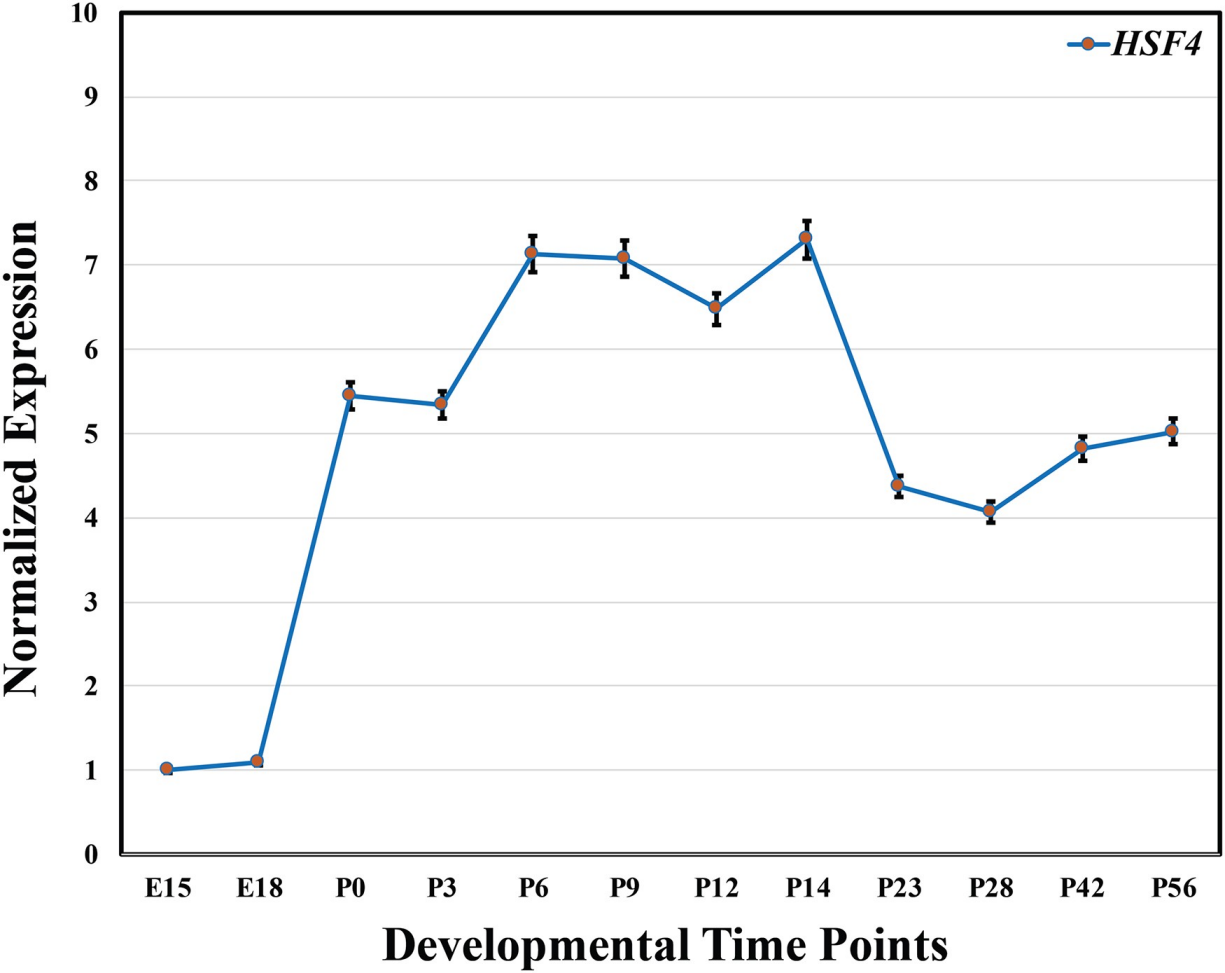
Expression pattern of *Hsf4* in mouse lens at different developmental time points. The expression levels were normalized with *Gapdh* and the expression of *Hsf4* at embryonic day 15 (E15) was chosen as a reference point to compare the relative expression at different developmental time points. The x-axis and y-axis represent developmental time points and relative expression of *Hsf4* mRNA, respectively.

Nakai and colleagues reported the expression of *HSF4* in multiple human tissues including heart, brain, skeletal muscle, and pancreas,[28] and we identified the expression of *HSF4* in our transcriptome and proteome studies.[34] We examined the expression of *Hsf4* in the embryonic and postnatal murine lens. As shown in Fig 4, we observed expression of *Hsf4* in mouse lens at embryonic days 15 and 18 (E15 & E18) that increased at birth, postnatal day 0 (P0). Subsequently, the expression of *Hsf4* remained at steady levels at P0 and over the remaining time course until two months of age (Fig 4).

**Fig 5:**
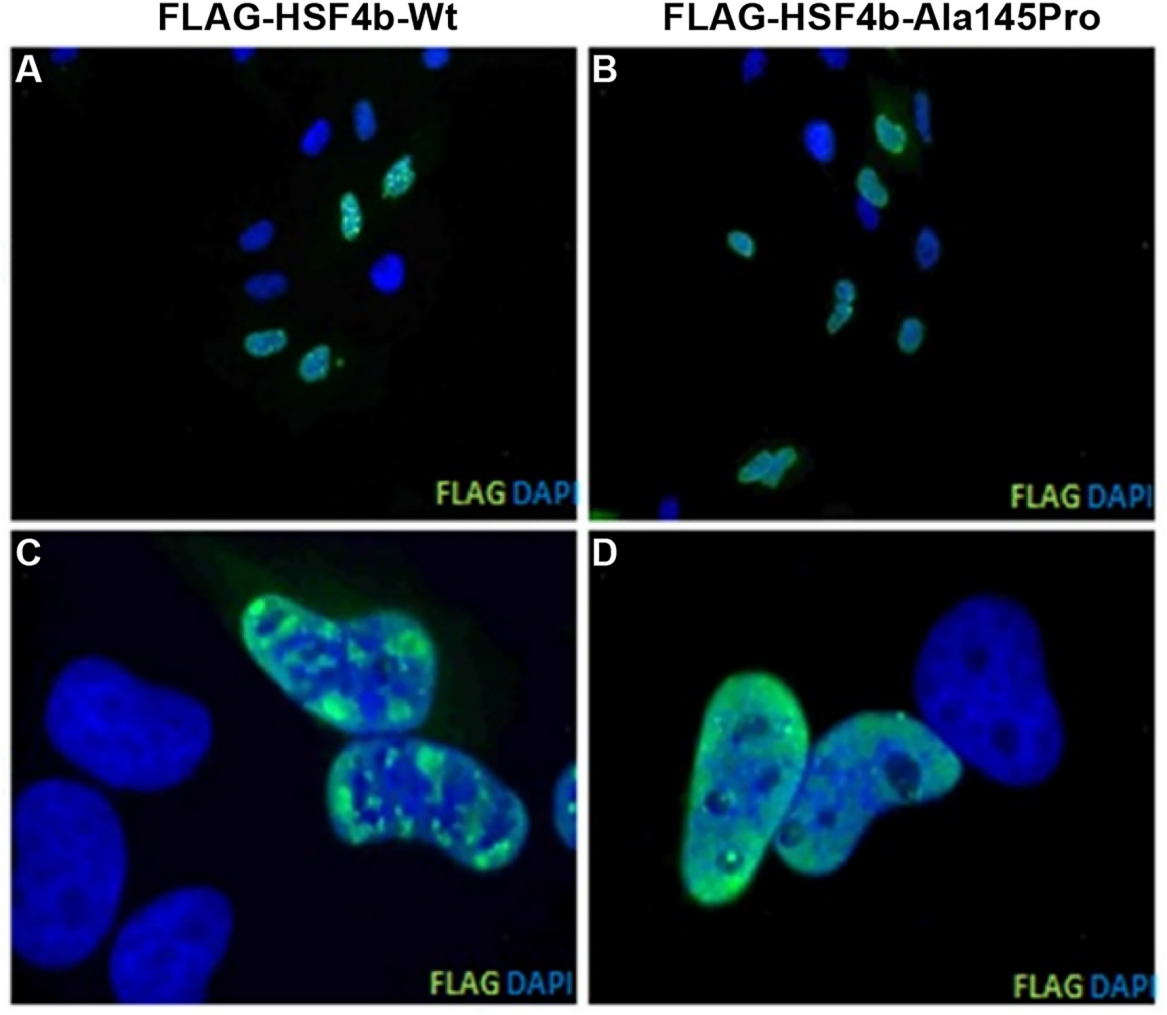
Sub-cellular localization of wild-type and mutant HSF4b (p.Ala145Pro) in HeLa cells. The cells were transfected with wild-type (A, C) and mutant HSF4b (B, D) constructs fused with C-terminus FLAG. The monoclonal anti-FLAG antibody was used to detect the FLAG-tagged wild-type and mutant HSF4b proteins. Nuclei were stained with DAPI. The wild-type (panels A and C) and mutant HSF4b (panels B and D) illustrate a nuclear localization pattern. The panels (A and B) and (C and D) represent 20× and 100× magnifications, respectively.

HSF4, a heat-shock transcription factor has been shown to primarily localized in the nucleus.[29] To investigate the trafficking pattern, we transfected HeLa cells with FLAG-tagged wild-type or mutant HSF4b constructs and tracked the localization of the protein with anti-FLAG antibody. The immunofluorescence tracking illustrated that both wild-type and mutant HSF4b proteins localized to the nucleus in HeLa cells (Fig 5). These results indicate that the missense mutation (p.Ala145Pro) does not affect the nuclear transportation of the mutant protein. However, we identified a diffused nuclear distribution pattern of the mutant protein, in contrast to the accumulation of wild-type protein as speckles in the nucleus (Fig 5).

## Discussion

Here, we report a novel causal mutation in *HSF4* associated with autosomal recessive congenital cataracts in a consanguineous Pakistani family. The slit-lamp ophthalmic examination confirmed nuclear cataracts in PKCC074, while genome-wide scan localized the critical interval to chromosome 16q with a maximum two-point LOD score of 4.51 at θ = 0. Sequencing of the coding exons of *HSF4* identified a novel missense mutation that segregated with the disease phenotype in the family and was absent in control chromosomes. Taken together, these results strongly suggest that mutation in *HSF4* is responsible for recessive congenital cataracts in PKCC074.

**Fig 6:**
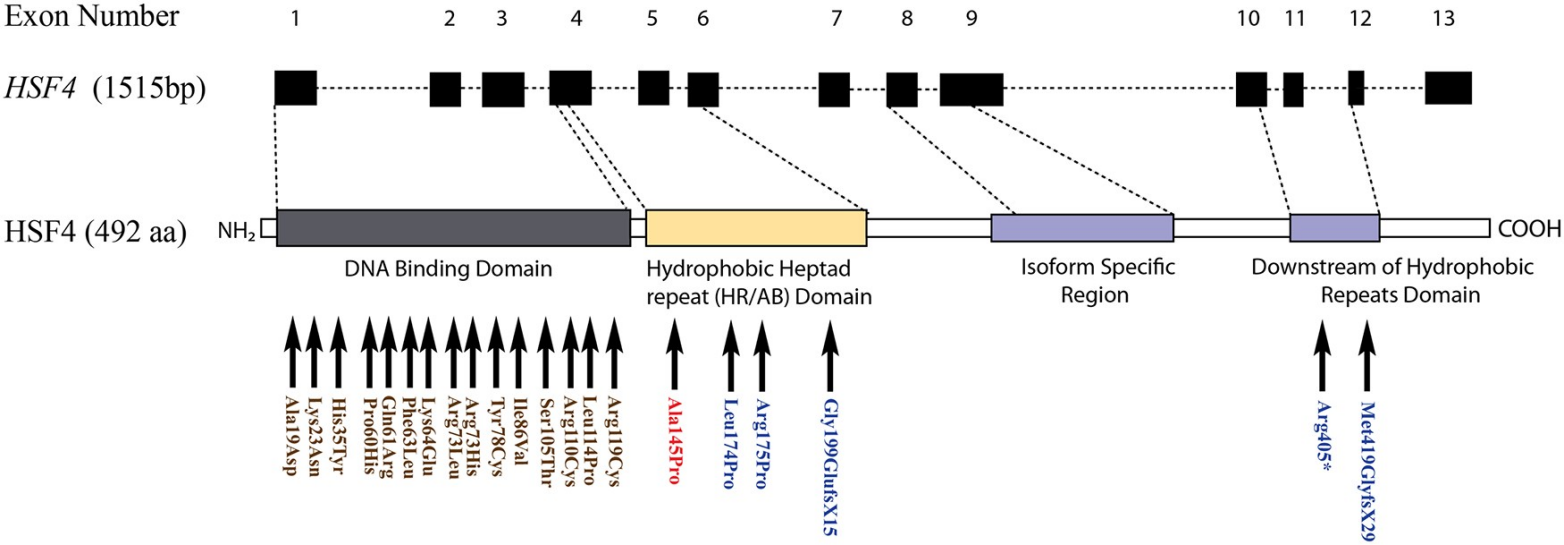
A schematic representation of the protein structure and causal variants identified in *HSF4*. Mutations previously identified in *HSF4* responsible for autosomal dominant cataracts are shown in brown, mutations responsible for autosomal recessive cataracts are shown in blue. The p.Ala145Pro mutation shown in red was identified in the study. **Note**: In addition to variants presented in the figure, a splice site variant (IVS5 c.233-1G>A) in HSF4 has been reported for autosomal dominant cataracts by Cao and colleagues [38].

To date, 21 different mutations in *HSF4* have been reported with 16 of them associated with autosomal dominant and five with autosomal recessive cataracts (Fig 6).[35] So far, all the dominant mutations are single base substitutions residing in the DNA-binding domain of HSF4 with the exception of the splice-site mutation (Fig 6). In contrast, the recessive mutations are located in hydrophobic repeats (HR-A/B) or downstream of the hydrophobic repeat, important for a trimeric formation and transcriptional activation of HSF4.[33] HSF4 mutant alleles responsible for dominant and recessive cataracts reveal a distinct location pattern, suggesting that a single mutant allele in the DNA binding domain is sufficient for cataractogenesis, whereas, homozygous mutant alleles residing outside of DNA binding domain are required for cataract phenotype. Among five mutations associated with autosomal recessive cataracts, three mutations (G199EfsX15, R405X, and M419GfsX29) result in a truncated HSF4 protein. Merath and colleagues demonstrated that all three truncated HSF4 proteins localized to the nucleus; however, they failed to activate HSE-mediated luciferase reporter activity suggesting that loss of the HSF4 transcriptional activity may be responsible for cataractogenesis.[33]

Additionally, Forshew and colleagues reported a missense mutation (p.Arg175Pro) associated with autosomal recessive congenital cataracts.[36] In contrast to the above mentioned three recessive mutations, the p.Arg175Pro mutant allele encodes for a 492-amino acid full-length protein with single amino acid substitution.[36] The mutation resides within the hydrophobic heptad repeats (HR-A/B) domain and it has been suggested that causal mutation interferes with HSF4 trimerization and indirectly affects the DNA-binding activity of mutant HSF4.[37] Likewise, the mutation (p.Ala145Pro) identified in our study resides with the (HR-A/B) domain and given the fact that amino acid Proline is notorious for distorting the native protein structures, it is tempting to speculate that the causal mutation identified in PKCC074 affects the trimerization of HSF4. We identified a similar localization pattern for the wild-type and mutant HSF4b, confirming the nuclear localization of the mutant HSF4 protein. However, mutant protein exhibits a diffused nuclear distribution compared to the accumulation of wild-type protein as speckles in the nucleus. Thus, it could be reasoned that the diffused nuclear localization affects the binding of mutant HSF4 protein to the promoter and may be responsible for cataractogenesis observed in PKCC074.

In conclusion, here, we report a novel missense mutation in *HSF4* responsible for autosomal recessive congenital cataracts in a large consanguineous familial case. Identification of a second missense mutation in the HR-A/B domain will help us to better understand the role of HSF4 in ocular lens morphogenesis and particularly in the maintenance of lens transparency.
